# Supplementary material for: Ammonium Ion Enhanced V2O5-WO3/TiO2 Catalysts for Selective Catalytic Reduction with Ammonia
Source: Nanomaterials (Basel). 2021 Oct 12;11(10):2677. doi: 10.3390/nano11102677 (PMC8540173; doi:10.3390/nano11102677)
Supplement: Supplementary file 1 [file nanomaterials-11-02677-s001.zip › nanomaterials-1382125-supplementary.pdf]

Supplementary Materials

Ammonium Ion Enhanced V_2O_5-WO_3/TiO_2 Catalysts for Selective Catalytic Reduction with Ammonia

Min Seong Lee [1,2,†], Sun-I Kim [1,†], Bora Jeong [1], Jin-Woo Park [2,3], Taehyo Kim [1], Jung Woo Lee [2], Gibum Kwon [4,*] and Duck Hyun Lee [1,*]

1 Green Materials & Processes R&D Group, Korea Institute of Industrial Technology, Ulsan, 44413, Korea

2 Department of Materials Science & Engineering, Pusan National University, Busan 46241, Korea

3 NANO. CO., Ltd., Sanju 37257, Korea

4 Department of Mechanical Engineering, University of Kansas, Lawrence, Kansas 66045, USA

* Correspondence to: dulee@kitech.re.kr (D.H.L.), gbkwon@ku.edu (G.K.)

† Both authors contributed equally to this work.

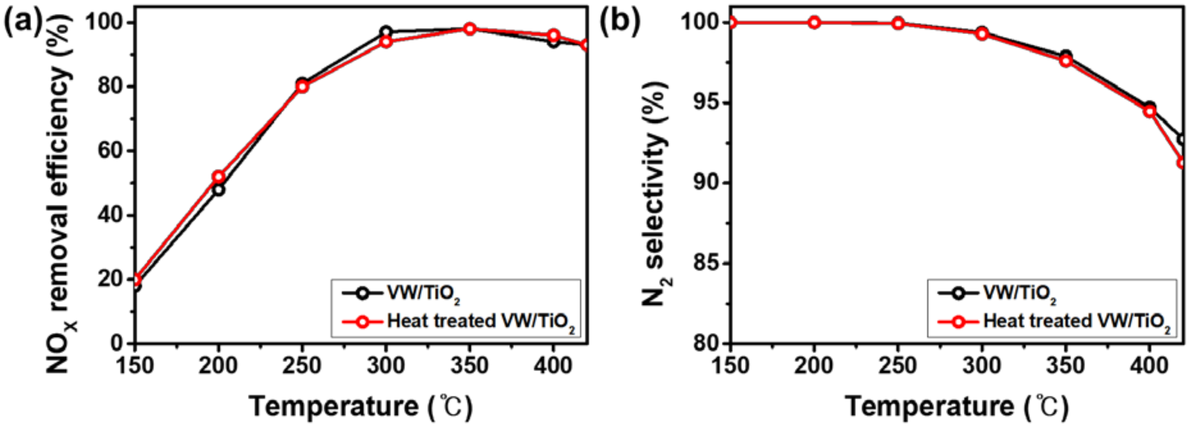

Figure S1. NO_x removal efficiency (**a**) and N_2 selectivity (**b**) of V_2O_5–WO_3/TiO_2 (black line) and heat-treated V_2O_5–WO_3/TiO_2 without the silane coating process (red line). Reaction conditions: [NO] = 300 ppm, [NH_3] = 300 ppm, [SO_2] = 600 ppm, [O_2] = 5 vol.%, and GHSV = 60,000 h^{-1}. There was no significant differences in the NOx removal efficiency and N_2 selectivity between V_2O_5–WO_3/TiO_2 and heat-treated V_2O_5–WO_3/TiO_2 without the silane coating process in the temperature range of 150–420 °C. These results demonstrate that silane coating is a key process in preparing ammonium ion-enhanced V_2O_5–WO_3/TiO_2 catalysts.

Table S1. Atomic percent of elements obtained from the XPS spectra of the heat-treated VW/TiO_2 catalyst without silane coating process.

Sample	Elemental Composition (at. %)								
	N	O	Si	F	C	S	V	W	Ti
VW/TiO_2	0.40	52.47	-	-	18.28	0.53	1.55	1.82	24.95
Heat-treated VW/TiO_2 (without silane coating)	0.27	55.27	-	-	19.79	0.36	1.29	1.55	21.47

Table S2. X-ray fluorescence analysis of the commercial plate-type monoliths.

Sample	Chemical Composition (wt. %)					
	TiO_2	V_2O_5	WO_3	MoO_3	Al_2O_3	CaO
Commercial plate-type monoliths	91.08	1.88	0.46	2.15	2.04	1.41
